# Inhibitory targeting cGAS-STING-TBK1 axis: Emerging strategies for autoimmune diseases therapy

**DOI:** 10.3389/fimmu.2022.954129

**Published:** 2022-09-12

**Authors:** Min Zhang, Yan Zou, Xujun Zhou, Jinming Zhou

**Affiliations:** ^1^ Key Laboratory of the Ministry of Education for Advanced Catalysis Materials, Department of Chemistry, Zhejiang Normal University, Jinhua, China; ^2^ Drug development and innovation center, College of Chemistry and Life Sciences, Zhejiang Normal University, Jinhua, China

**Keywords:** CGAS, STING, autoimmune disease, inhibitor, TBK1

## Abstract

The cGAS-STING signaling plays an integral role in the host immune response, and the abnormal activation of cGAS-STING is highly related to various autoimmune diseases. Therefore, targeting the cGAS-STING-TBK1 axis has become a promising strategy in therapy of autoimmune diseases. Herein, we summarized the key pathways mediated by the cGAS-STING-TBK1 axis and various cGAS-STING-TBK1 related autoimmune diseases, as well as the recent development of cGAS, STING, or TBK1 selective inhibitors and their potential application in therapy of cGAS-STING-TBK1 related autoimmune diseases. Overall, the review highlights that inhibiting cGAS-STING-TBK1 signaling is an attractive strategy for autoimmune disease therapy.

## 1 Introduction

Autoimmune diseases including various chronic inflammatory illnesses have affected the health of around 3%-10% of people in the world (1). The aberrant responses of the immune system to self are thought as the major factor leading to autoimmune diseases. Although the innate immune system which detects and responds to the pathogen-associated molecular patterns (PAMPs) and damage-associated molecular patterns (DAMPs) serves as the organism’s first line of defense against foreign invasion, the dysregulation and over-activation of the innate immune system will lead to various inflammatory illnesses (2, 3). Moreover, the autoinflammation induced by the abnormal innate immune signaling can achieve the establishment of adaptive immune responses, thus leading to the progress of autoimmunity. The endosomal or cytosolic nucleic-acid sensing involved in innate immunity is one of the initial triggers of autoimmunity. The nuclear acid recognition receptors, including retinoic acid-inducible gene I (RIG-I), melanoma differentiation-associated gene 5 (MDA5), Toll-like receptors (TLR3, 7, 8, and 9), and the cyclic GMP-AMP synthase–stimulator of interferon genes–tank-binding kinase 1 (cGAS-STING-TBK1) axis, have been directly related to the pathogenesis of various autoimmune diseases (4–9).

The cGAS-STING signaling pathway combines DNA sensing with the induction of a strong innate immune defense program, playing a crucial role in the host immune response (10). Through the recognition of the exogenous DNA from virus and bacterial or own damaged DNA, cGAS catalyzes the synthesis of cyclic GMP-AMP (cGAMP) from adenosine triphosphate (ATP) and guanosine triphosphate (GTP). cGAMP further interacts with STING and activates downstream pathways to induce the expression of type I interferons (IFNs), interferon-stimulator genes (ISGs), and other pro-inflammatory cytokines, thus extensively activating the host immune system and further inhibiting and eliminating tumors or viruses (7, 11–14). The dysregulation of this broad and powerful recognition system (cGAS-STING) can also disrupt the dynamic homeostasis of cells and organs by inducing aberrant innate immune responses and a variety of inflammatory triggers (15, 16). The persistent or chronic inflammatory signaling that links to the activation of cGAS-STING signaling is prone to developing the autoimmune disease including Aicardi-Goutières syndrome (AGS), systemic lupus erythematosus (SLE), STING-associated vasculopathy of infancy (SAVI), and amyotrophic lateral sclerosis (ALS), etc. (17–21) Interestingly, the cGAS-STING pathway inhibitors such as H-151 effectively improved the symptoms of the autoimmune diseases ALS and psoriasis by decreasing the inflammatory signaling in animal models, thereby becoming a promising therapeutic agent for autoimmune diseases (17, 22). Moreover, Ablasser’s lab reported that H-151 inhibited the inflammation in SARS-COV-2 driven disease COVID-19 (23). Herein, we focus on the key pathways mediated by cGAS-STING and various cGAS-STING-TBK1 signaling related autoimmune diseases, as well as the recent development of cGAS, STING, or TBK1 selective inhibitors.

## 2 Basic structural features of cGAS-STING-TBK1 axis

### 2.1 The structural features of cGAS

**Figure 1:**
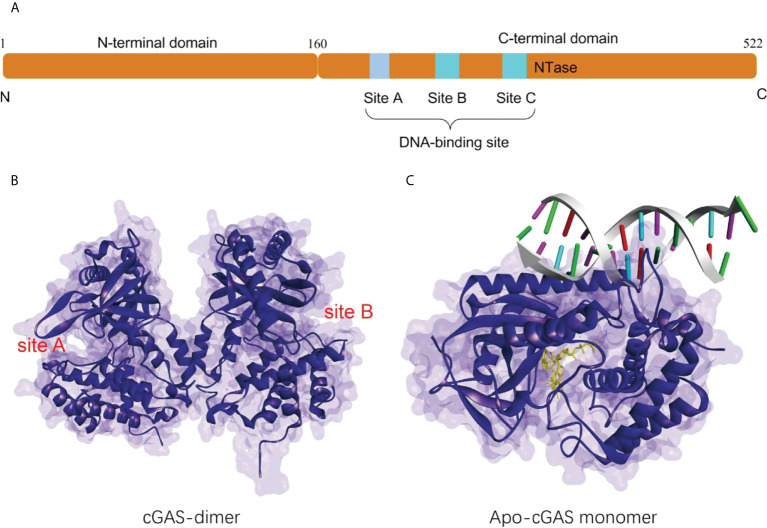
**(A)** Schematic organization of the structural domain of human cGAS; **(B)** Structure of the human cGAS dimer (PDB-ID: 4LEV); **(C)** Structure of porcine cGAS (blue ribbon, apo-cGAS monomer) in complex with DNA, ATP and GTP (yellow ribbon, PDB ID: 4KB6).

Human cGAS is a 60 kD protein from the nucleotidyl transferase (NTase) family, consisting of a non-conserved N-terminal structural domain (1-160) and a highly conserved C-terminal NTase structural domain (161-522) (**Figure 1A**) (24). The N-terminal domain is identified to play a role in stabilizing or suppressing cGAS protein (25).While the NTase domain contains three dsDNA binding sites and is essential for dsDNA recognition and the synthesis of the second messenger 2’3’-cGAMP. The cGAS can bind non-sequence-dependently to dsDNA through the phosphate backbone, leading to significant conformational changes in the NTase structural domain of cGAS and a structural switch in the catalytic pocket, which initiates the catalytic synthesis of GTP and ATP to 2’3’-cGAMP (**Figures 1B, C**) (10, 15, 26). The synthesis of 2’3’-cGAMP is a critical step in the triggering of the STING-mediated immune system (14, 27). The binding of long-stranded dsDNA to cGAS which forms a ladder-like network is thought necessary to the activation of the cGAS-STING signaling pathway, thus becoming a pattern that effectively prevents the activation of STING by the short-stranded DNA (28, 29). cGAS binds to the negatively charged acidic patch formed by histones H2A and H2B through its DNA binding site. High-affinity nucleosome binding prevents dsDNA recruitment and keeps cGAS in an inactive conformation (30–34).

### 2.2 The structural features of TBK1

**Figure 2:**
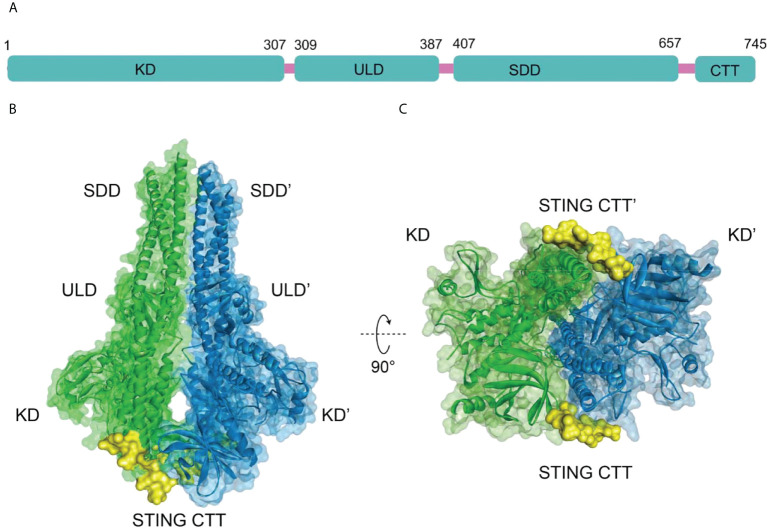
**(A)** Schematic organization of the structural domain of human TBK1, **(B)** Structure of human TBK1 in complex with chicken STING CTT (PDB-ID: 6NT9); **(C)** Bottom view of TBK1 structure.

The human TBK1 kinase consists of 729 amino acids, including an N-terminal kinase domain (KD), a ubiquitin-like domain (ULD), an alpha-helical scaffold dimerization domain (SDD), and a C-terminal adaptor-binding domain (CTD) (**Figure 2A**) (35, 36). Extensive interactions between KD, ULD, and SDD form the dense TBK1 dimer. The KD of TBK1 consists of N-terminal and C-terminal leaflets with an active ATP binding site at the interface. Ser172 residue on the activation loop is the phosphorylation site for TBK1 kinase (37). When TBK1 is phosphorylated, the αC-helix of the kinase structural domain rotates to the inward active position, facilitating the formation of a critical salt-bridge interaction between Glu55 of the αC-helix and Lys38 at the active site. However, when TBK1 is in the inactive conformation, the activation loop is disrupted and the αC-helix is positioned in an inactive position outside the ATP-binding structural domain (35). In the structure of the TBK1 dimer, the activation of TBK1 is mainly controlled by trans-autophosphorylation, in which two KDs limit the cis-autophosphorylation activity of TBK1 (38). A highly conserved PLRT/SD motif in the C-terminal tail (CTT) of STING mediates the recruitment of TBK1 by binding directly to the dimeric interface of TBK1. Further analysis of the crystal structure of STING and TBK1 showed that the dimeric TBK1 binds to two monomers of the CTT of STING, with each STING monomer simultaneously binding to two TBK1 monomers to form a 2:2 complex (**Figures 2B, C**) (39). The 2’,3’-cGAMP binding initiates the STING activation by forming a stable oligomer, and the conserved PLPLRT/SD protein motif in STING-CTT can dimerize the TBK1 interface to induce the phosphorylation and activation of STING and TBK1 through hydrophobic binding. Further recruitment and phosphorylation of interferon regulatory factor 3 (IRF3) and TBK1 leads to the involvement of downstream signaling components and the inducible regulation of IFN-I transcription, which is the hallmark signal for the initiation of the cGAS-STING-TBK1 signaling pathway (35, 39–42).

### 2.3 The structural features of STING

**Figure 3:**
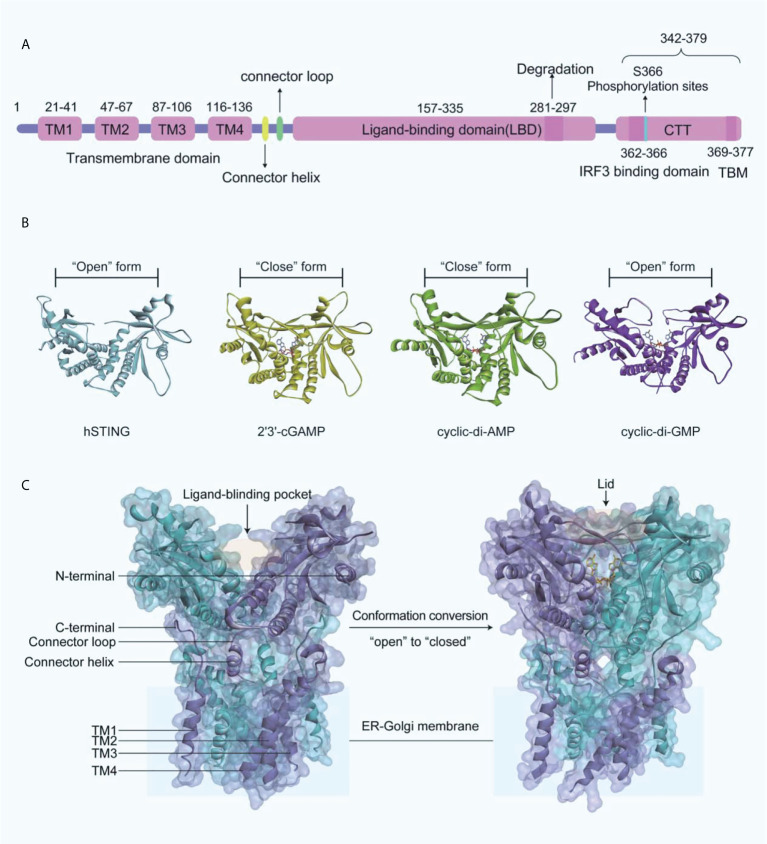
**(A)** Schematic organization of the structural domain of human STING; **(B)** Crystal structures of human STING CTD (blue ribbon, apo-STING, PDB-ID: 4EMU; yellow ribbon, STING bound 2’3’-GAMP, PDB-ID: 4LOH; green ribbon, STING bound CDA, PDB-ID: 4F5D; purple ribbon, STING bound CDG, PDB-ID: 4F5Y); **(C)** On the left is the crystal structure of full-length human STING in open conformation (PDB-ID: 6NT5), and on the right is the crystal structure of chicken STING and 2’3’-cGAMP in closed conformation (PDB-ID: 6NT7).

Human STING (MITA) is a transmembrane protein located on the endoplasmic reticulum (ER) and consists of an N-terminal transmembrane structural domain (NTD) containing four transmembrane helices TM1 (residues 21-41), TM2 (residues 47-67), TM3 (residues 87-106), TM4 (residues 116-136) and a globular C-terminal structural domain (CTD, residues 157-379) (**Figure 3A**). STING is highly expressed in immunomodulatory-related cells and tissues such as bone marrow, spleen, and peripheral blood leukocytes (43–46). The STING-CTD is comprised of a ligand-binding domain (LBD, residues 157-335), an IRF3-binding domain (residues 362-366), and a TBK1-binding motif (TBM, residues 369-377) (47–49). Through biophysical technology, especially X-ray crystallography, the STING-CTD is identified as a butterfly-like dimer with a ligand binding site located at the groove of the interface (**Figure 3B**).

The endogenously produced 2’3’-cGAMP is detected by STING, which in turn binds to STING CTD in a dimeric form (45). Subsequently, STING performs extensive conformational changes, with an inward 180°C flip of the V-type STING LBD dimer, an “open” to “closed” transition of conformation to form a “lid” covering the 2’3’-cGAMP binding site (**Figure 3C**) (45, 50). The formation of STING polymers via a C148-mediated disulfide bond is essential to the activation of STING, while the “opening” or “closing” of the LBD regulates its activity by affecting the affinity with the ligand to the protein (50). It is important to note that cyclic-di-GMP (CDG) binding does not produce the conformational changes induced by 2’3’-cGAMP or cyclic-di-AMP (CDA), while CDG-bound STING may also lead to the activation of the cGAS-STING-IRF3 pathway (**Figure 3B**). What is more, recent research indicated that the potent STING agonist diABZI did not promote the closure of the lid region of STING either (51).

The subsequent translocation of STING from the ER to the Golgi apparatus is mediated primarily by coat protein complex II (COPII) vesicles (52), which is dependent on GTPase SAR1A and COPII complex components, including SEC24C and ARF-GTPase ARF1. After transporting to the Golgi apparatus, STING is palmitoylated at two cysteine residues Cys88 and Cys91, which is necessary for the recruitment of TBK1 and IFNs transcription (53, 54). However, TBK1 recruiting to STING alone does not induce the activation of IRF3 at the CTT of STING (residues 342-379) (**Figure 3A**). The residues Leu333 and Arg334 at STING-CTD play critical roles in c-GAMP-induced autophagy and phosphorylation of TBK1 and IRF3 (52). TBK1 phosphorylates IRF3, which subsequently induces the dimerization and translocates into the nucleus, thereby driving the transcriptional expression of IFNs (55). After the initiation of downstream signaling, STING is degraded in endolysosomes, and the residues 281-297 are required for the transport-mediated STING degradation (56).

## 3 cGAS-STING-mediated signaling pathways

### 3.1 cGAS-STING-IRF3 pathway

**Figure 4:**
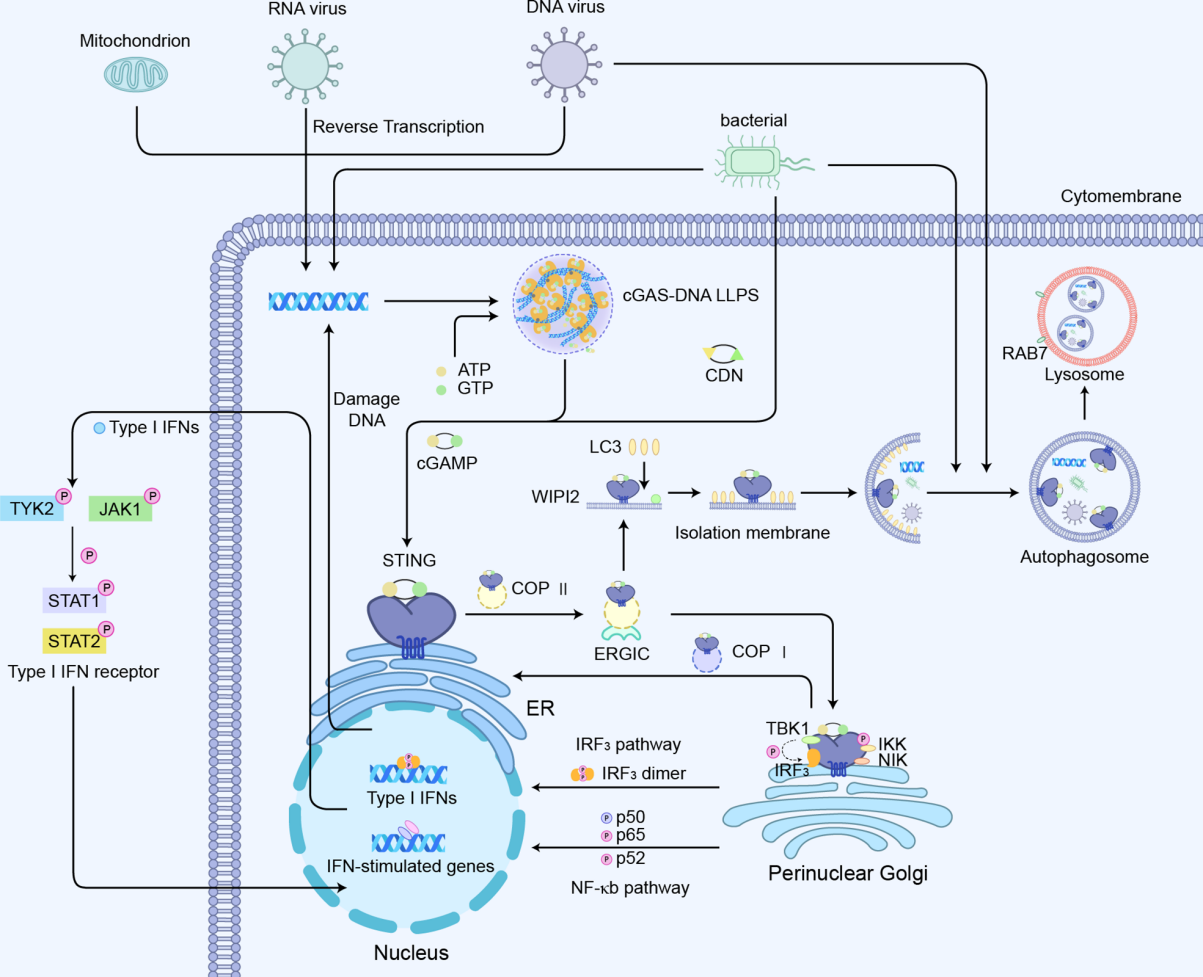
cGAS-STING-mediated signaling pathways:1) cGAS-STING-IRF_3_ pathway; 2) cGAS-STING mediated NF-κb pathway activation; 3) cGAS -STING induced autophagy process.

The activation of the cGAS-STING axis will induce the modification of IRF3 and its translocation to the nucleus, thereby driving the transcriptional expression of IFNs (57–59). Simultaneously, the binding of IFNs to its receptor activates Janus kinases (JAKs), including JAK1 and tyrosine kinase 2 (TYK2), which in turn phosphorylate the receptor (20). This process allows DNA-binding protein signal transducer and activator of transcription 1 (STAT1) and 2 (STAT2) to bind to the receptor, thereby phosphorylating and dimerizing them. The dimer then translocates to the nucleus where it upregulates the transcription of IFN-responsive genes, including the transcription of IFNs dependent on interferon regulatory factor 9 (IRF9) (**Figure 4**). The synthesis and release of IFN and its binding to the IFN receptor further upregulated the interferon genes in a positive feedback loop (20).

To avoid a severe inflammatory response to the induced transcription of excess IFN, there is also an associated negative feedback mechanism. When the cGAS-STING-mediated immune response is continuously activated, STING is induced to be degraded in the endosome. Following the activation and translocation of STING, it is phosphorylated by serine/threonine protein kinase 1/autophagy-associated protein 1 (ULK1/ATG1) to inhibit the sustained induction into IFNs and inflammatory disease (60). ULK1-2 function can be regulated by AMP-activated protein kinases (AMPK) or mammalian targets of rapamycin (mTOR) which is activated under cellular stress conditions. Cytoplasmic dsDNA and/or CDN are found to activate ULK1-2, which initiates a negative feedback loop controlling STING overexpression through restricting STING translocation from the Golgi apparatus by autophagy-associated protein 9a (ATG9a) and decreasing the association of STING and TBK1 (61). NOD-like receptor C3 (NLRC3) binds to STING and prevents its translocation from the ER to the Golgi apparatus, thereby reducing the IFNs response (62). Also, the movement of STING outside the ER facilitates its recruitment to LC3 autophagic vesicles through a WD repeat structural domain phosphoinositides interacting protein 2 (WIPI2)-dependent mechanism (52). LC3 coordinates the negative regulation of STING by transporting STING complexes, DNA, and pathogens to autophagy vesicles for lysosomal dependent degradation, a process that requires the RAS-associated protein Rab-7a (RAB7) GTPase (52, 63). Moreover, recent findings have revealed a mechanism that moves STING from the Golgi to the ER to downregulate the cellular activation of STING. Specifically, the adaptor protein SURF-4 interacts with STING on the Golgi apparatus to promote STING encapsulation into coat protein complex I (COPI) vesicles for retrograde transporting STING from the Golgi apparatus to the ER, thereby inhibiting sustained STING activation (64, 65). Upon the entry of IRF3 into the nucleus and the activation of ISGs, the STING-TBK1-IRF3 complex is dissociated and drives E3 ubiquitin ligase RNF5/TRIM30α mediated K48-linked polyubiquitinated STING, which promotes the degradation of STING via the proteasome pathway (66, 67).

### 3.2 cGAS-STING mediated NF-κb pathway activation

Another major signaling module involved in the regulation of STING is nuclear factor kappa B (NF-κB)-mediated transcriptional activation, which promotes the expression of several pro-inflammatory cytokines such as TNF-α, IL-1β, and IL-6 (**Figure 4**) (43). The CTT motif of STING is necessary for triggering the IRF3-dependent transcription of IFNs, whereas the STING-dependent NF-κB pathway is not entirely relied on the CTT of STING (68). STING-mediated NF-κB activation indicates much less sensitivity to the knockout of TBK1 (69). TBK1 alone is dispensable for STING-induced NF-κB responses in human immune cells, while acts redundantly with IκB kinase ϵ (IKKϵ) to drive NF-κB upon STING activation (68, 70). Consistently, the ancestral STING homologs in insects and early postlarvae completely lack CTT signaling, but could still achieve a host defense by promoting NF-κB responses. Using a tamoxifen-induced TBK1 deficiency model in adult mice, it was observed that TBK1 deficiency had little effect on cytokines of NF-κB following the administration of the mouse STING agonist DMXAA (69). Interestingly, the nuclear DNA damage would induce the non-canonical activation of STING by ATM and IFI16, leading to the activation of NF-κB signaling (71). Also, the genotoxic DNA damage induced by camptothecin drove IL-6 production through non-canonical STING signaling in the STING-expressing cancer cells (72). The stimulation of cGAS-STING also promotes a non-canonical NF-κB response by triggering p52 nuclear translocation (**Figure 4**) (73, 74). This signaling restricts IFN-I and the classic NF-κB pathway as regulators of the negative feedback mechanism of STING (75). Therefore, the explicit mechanisms of STING interacting with NF-κB pathway components are still required to be verified.

### 3.3 cGAS -STING-induced autophagy process

Previous work suggested that autophagy induction via STING trafficking is a primal function of the cGAS-STING pathway (52). The cGAS–STING pathway can induce canonical autophagy through liquid-phase separation of the cGAS–DNA complex, the interaction of cGAS and Beclin-1, and STING-triggered ER stress–mTOR signaling. Moreover, both cGAS and STING can trigger non-canonical autophagy via LC3-interacting regions and binding with LC3. What is more, autophagy induced by the cGAS–STING pathway plays crucial roles in balancing innate immune responses, maintaining intracellular environmental homeostasis, and restricting tumor growth (76). Conventional autophagy dependent on the ULK complex and TBK1 is involved in STING-mediated LC3 autophagy vesicle formation, and the activation of STING can also trigger non-canonical autophagy responses mediated by the PI3P effector WIPI2 and the ATG5-12-16L1 complex (**Figure 4**) (52, 60, 61, 77, 78). STING activation is indispensable for autophagic induction LC3 interacting regions (LIRs), and mutants of STING abolish its interaction with LC3 and its activation of autophagy (78). Of note, autophagy components also feedback on the regulation of STING activity through assisting STING intracellular trafficking capabilities as well as its lysosomal degradation (79). The direct interaction between Beclin-1 autophagy protein and cGAS not only inhibits STING signaling and decreases IFNs expression but also promotes the autophagy-mediated degradation of cytosolic DNA to avoid excess cGAS-STING activation (80). Similarly, key genes involved in the mechanism of autophagy, such as ULK-1 and Atg9, have appeared to suppress the STING/TBK1/IRF3 pathway, effectively inhibiting sustained immune response and excessive inflammation (60, 61). Moreover, autophagy receptor CCDC50 modulates STING-directed IFNs signaling activity by delivering the K63-polyubiquitinated STING to autolysosomes for the degradation (81).

## 4 cGAS-STING related autoimmune diseases

### 4.1 Monogenic autoinflammatory syndromes

#### 4.1.1 Aicardi-goutières syndrome

**Figure 5:**
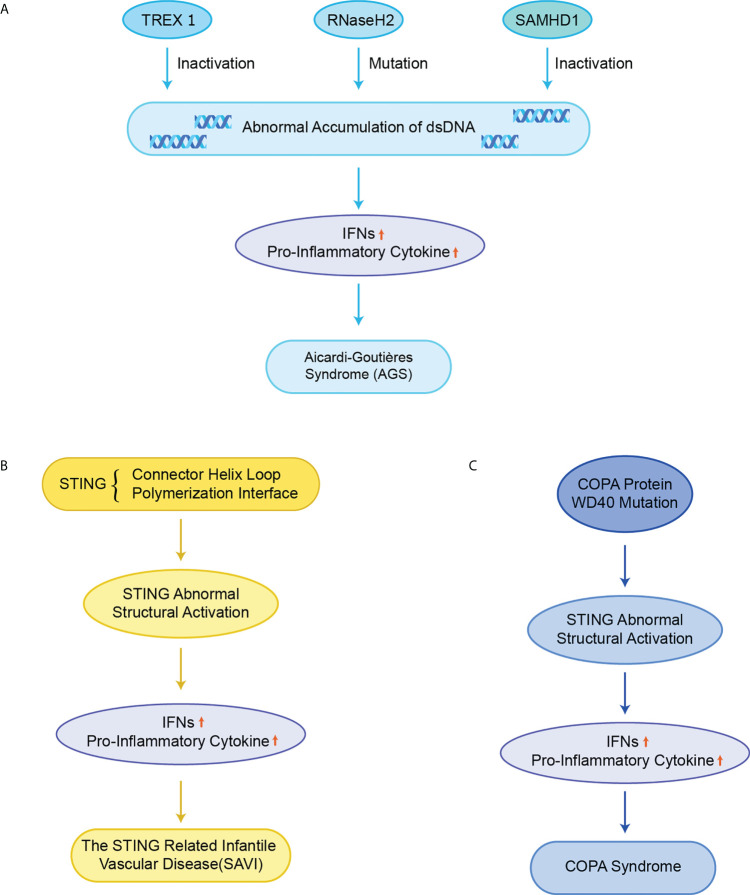
STING in monogenic autoinflammatory syndromes **(A)** Inactivation of TREX1, RNaseH2 and SAMHD1 leads to abnormal accumulation of dsDNA in normal cells, which over-activate the cGAS-STING signaling pathway, upregulates the expression of IFNs and pro-inflammatory cytokines, and ultimately triggers AGS; **(B)** Mutations in STING in the connector helix loop (N154S, V155M, and V147L) and the polymerization interface (G207E, R281Q, R284G, and R284S) lead to structural activation of STING, upregulating the expression of IFNs and pro-inflammatory cytokines, and ultimately causing SAVI; **(C)** Missense mutations in the structural domain of COPA WD40 impair endoplasmic reticulum binding and target protein sequencing, leading to structural activation of STING, upregulating the expression of IFNs and pro-inflammatory cytokines, and ultimately causing COPA syndrome.

AGS is an early-onset systemic inflammatory disorder that manifests clinically as neurological dysfunction and frostbite-like skin lesions. The nuclei acid exonuclease TREX1 was the first gene found to be related to AGS (**Figure 5A**) (82). TREX1 prevents excessive accumulation of endogenous auto-DNA and prevents aberrant activation of DNA-mediated cGAS-STING signaling, while structurally inactivated TREX1 leads to the IFN-dependent autoimmune disease AGS (19). In addition, it has also been reported that mice with mutations in three RNaseH2 enzyme complexes (RNaseH2 A, RNaseH2 B, and RNaseH2 C) exhibit increased IFN signaling and inflammation, and ultimately cause AGS-like symptoms (83).The failure of mutated RNaseH2 to degrade RNA/DNA hybrids led to the excessive activation of cGAS-STING signaling, which induced AGS. The lethality of some mice with dysfunctional mutated RNaseH2 was rescued by the knockout of STING (84). Similarly, sterile α motif and histidine-aspartate domain-containing protein 1 (SAMHD1) promotes the degradation of nascent DNA in human cell lines by stimulating the exonuclease activity of meiotic recombination 11 homolog A (MRE11A), and the deletion of SAMHD1 lead to the accumulation of genomic DNA in the cytoplasm and triggers AGS (85).

#### 4.1.2 STING-associated vasculopathy of infancy

Mutations in exon 5 of STING lead to functional activation of STING, resulting in the excessive STING-induced IFN signaling, causing a disorder termed SAVI including recurrent fever, ulcerative skin lesions, vasculitis, and interstitial lung disease (**Figure 5B**) (18, 20). Mutant residues are located in two separate regions on STING, the connector helix loop (N154S, V155M, G158A, G166E, H72N, and V147M/L) and the polymerization interface (C206Y/G, G207E, F279L, R281Q/W, and R284G/S) (86–89). Mutations in the regions can spontaneously rotate around the connected helix loop by inducing the LBD allosteric activation, or by promoting the STING polymerization, thereby triggering the ligand-independent activation of STING (50, 58). An obvious feature of the mouse SAVI model is the severe lymphopenia and immunodeficiency due to the abnormal lymphocyte development and aberrant intrinsic T cells (90). However, it has likewise been reported that the inhibition of IFN signaling did not affect disease pathogenesis in the N153S STING mouse model of SAVI. Instead, the T-cell depletion protected N153S mice from lung disease progressions, which may explain why JAK inhibitors targeting the IFN-α receptor (IFNAR) are not always successful in the treatment of SAVI patients (91).

The Ca2+ sensor stromal interaction molecule 1 (STIM1) has been reported to be a promising target for the treatment of SAVI, where STIM1 directly interacts with STING and inhibits the transport of STING from the ER to the Golgi apparatus (92). A peptide ISD017 has been reported to block the activity of STING in vivo and improve the disease progression of a mouse model of lupus in a STIM1-dependent manner (93). The activity of three disease-associated STING variants, V147L, N154S, and V155M, also can be inhibited by STIM1 in part by blocking their translocation to the ERGIC. In the SAVI model, the activation of STING leads to cellular T-cell defects by modulating T cell proliferation and differentiation (90, 94), and plays a key role in the initiation and progression of SAVI (95).

#### 4.1.3 COPA syndrome

Pathogenic COPA variants can lead to immune dysregulation in Mendelian syndrome. COPA is a subunit of COPI that mediates STING from the Golgi apparatus to ER transport, and the dysfunction of the target thereby leads to the structural activation of STING (**Figure 5C**) (96). In a mouse model of COPA syndrome (CopaE241K/+), IFN-driven inflammation of the mice could be rescued through crossing with STING-deficient mice (STING1gt/gt). In addition, the embryonic lethality in Purex COPAE241K/E241K mice could be rescued by the knockout of STING (64). JAK inhibitors can improve clinical performance and IFN levels, but the effect is very limited (97). In mouse models with functionally acquired STING mutations, the development of lung lesions is dependent on T cells instead of IFN-I (98). This result may explain the poor therapeutic effect of JAK inhibitors in human COPA syndrome. In addition, the small molecule STING inhibitor H-151 has also been reported to improve the inflammation in COPA syndrome (99). The inhibition of STING has emerged as an efficient way for the treatment of COPA syndrome.

### 4.2 Autoimmune neurodegenerative diseases

While the association of STING with neurodegenerative diseases has been poorly investigated in previous studies, the activation of the immune system is a prominent feature of several neurodegenerative diseases, including Alzheimer’s disease (AD), Parkinson’s disease (PD), Huntington’s disease (HD), frontotemporal dementia (FTD), multiple sclerosis (MS), and ALS. Several recent studies have revealed the relationship between STING and autoimmune neurodegenerative diseases (100). Although IFNs are also produced by neurons and astrocytes, STING is mainly expressed in microglia to elicit the IFN responses in the brain (101, 102). In chronic neurodegenerative disease states, aberrantly activated STING signaling induces the expression of IFNs and increases the phenotype of microglia and astrocytes, thereby accelerating the development of neuroinflammation (103, 104).

#### 4.2.1 Amyotrophic lateral sclerosis

**Figure 6:**
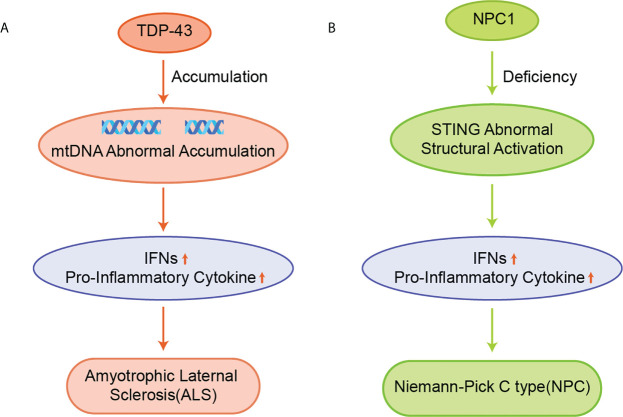
**(A)** Accumulation of TDP-43 leads to aberrant accumulation of mtDNA, which induces the expression of IFNs and pro-inflammatory cytokines in a cGAS/STING dependent manner and promotes the development of ALS; **(B)** NPC1 leads to the 2’3’-cGAMP-independent activation of STING, upregulating the expression of IFNs and pro-inflammatory cytokines, and ultimately causing NPC.

The cytoplasmic accumulation of TDP-43 is a hallmark of ALS (**Figure 6A**) (105). TDP-43 induces mtDNA release via voltage-dependent anion channel 1 (VDAC1), which subsequently induces IFNs and inflammatory cytokine expression in a cGAS-STING dependent manner (17). STING gene deletion or the use of small molecular inhibitors of STING significantly improved ALS symptoms and prolonged the life span of mice. Besides, the expansion of the hexanucleotide repeat sequence (GGGGCC) in the mice lacking chromosome 9 open reading frame 72 (C9orf72) gene is the most common cause of familial ALS (106). Dendritic cells isolated from mice lacking the C9orf72 protein showed marked early activation of IFN-I responses, and mice showed age-dependent lymphoid hypertrophy and autoinflammation. The C9orf72-deficient mice were more likely to develop experimental autoimmune encephalitis. Also, bone marrow cells lacking C9orf72 showed signs of hyper-activation upon being exposed to STING agonists and reduced autolysosomal degradation of STING. The C9orf72- deficient ALS patients had higher levels of IFN-I signaling than patients with sporadic ALS and could improve symptoms with STING inhibitors treatment.

#### 4.2.2 Niemann–pick disease type C

NPC is a chronic neuroautoimmune disease caused by a deficiency of Niemann-Pick C1 (NPC1), which leads to the impaired metabolism of neurospherin phospholipids (**Figure 6B**) (107). NPC1 is an auxiliary protein for transporting STING to the lysosome for its degradation. NPC1 deficiency leads to the accumulation of cholesterol and other lipids in the lysosome, resulting in the decrease of ER cholesterol levels and the activation of SREBP2-SCAP translocation from the ER to the Golgi apparatus (108). The STING protein is recruited by the SREBP2-SCAP complex, which triggers 2’3’-cGAMP-independent STING activation by hijacking STING to transport from the ER to the Golgi apparatus in NPC1 KO cells, thereby leading to a progressive loss of Purkinje in NPC1-/- mice, thus resulting in impaired motor function and reduced survival (63, 109).

#### 4.2.3 Multiple sclerosis

MS is considered a progressive autoimmune disease which is caused by inflammation and neurological damage from immune system attacks on myelin (110). Early studies reported that the antiviral drug ganciclovir induced the suppression of MS experimental autoimmune encephalomyelitis (EAE) model in a STING-dependent manner (109). STING acts as a regulator of microglial cell reactivity and neuroinflammation, which improves the pathology of EAE in mice by reducing immune cell infiltration and inhibiting the proliferation of microglia or the immune cells of the central nervous system. Besides, The oral administration of Bowman-Birk inhibitor (BBI), a serine protease inhibitor derived from soy was reported to inhibit EAE (111). The inhibition is dependent on STING and IFN-β secreted by macrophages, and the absence of IFNAR in bone marrow cells restricts the inhibition of EAE by BBI.

While it is true that neurodegenerative diseases occur after aberrant activation of the cGAS-STING pathway, the role of this pathway in different neurological diseases is still needed to be further investigated. The inhibition of the STING signaling may be a potential way in therapy of STING-associated neurodegenerative diseases. However, we must also note that activation of cGAS-STING signaling is not a single facilitator in neurodegenerative disease. IFNs, the downstream expression products are also negative regulators of some inflammation in the peripheral or central nervous system (109, 112). Therefore, how to effectively regulate the cGAS-STING signaling using a cGAS or STING modulator is key to the treatment of neurological autoimmune diseases.

### 4.3 Other cGAS-STING related autoimmune diseases

#### 4.3.1 Systemic lupus erythematosus

**Figure 7:**
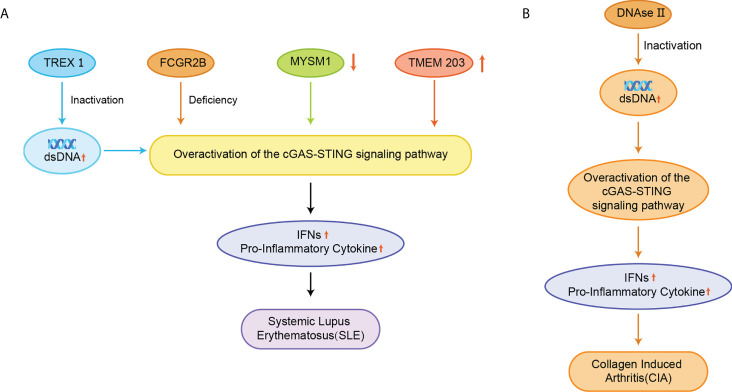
**(A)** Inactivation of TREX1 resulted in the accumulation of AdMVs in serum, deletion of the FCGR2B gene, decreased MYSM1 production in PBMC, and elevated TMEM203 signaling in T cells were associated with overexpression of the cGAS-STING signaling pathway, upregulation of IFNs and pro-inflammatory cytokine expression, and induction of SLE; **(B)** Inactivation of DNase II causes abnormal accumulation of dsDNA, which over-activates the cGAS-STING signaling pathway and triggers CIA.

SLE is also an autoimmune disease that has been reported to be related to STING. The elevated serum 2’3’-cGAMP levels in SLE, leading to the redundant STING activation, have been reported in approximately 15% of all SLE patients (21). The exact cause of SLE is not clear yet, but elevated dsDNA levels were identified in cells from SLE patients, and apoptosis-derived membrane vesicles (AdMVs) in the serum of SLE patients had high inducing ISGs (**Figure 7A**) (113). Defective clearance of apoptotic cells produces dsDNA-containing AdMVs, which in turn induces ISGs via the cGAS-STING pathway. Next, ISGs activate an immune response that leads to tissue damage in various organs, resulting in further production of AdMVs and a positive feedback loop of ISGs.

Besides, a comprehensive genetic analysis has identified FCGR2B as a susceptibility gene in patients with SLE, and the mutation of the FCGR2B gene leads to the induction of SLE (**Figure 7A**) (114). The disruption of STING signaling relieves the lupus development in FCGR2B-deficient mice, and the transplantation of STING-activated bone marrow-derived dendritic cells into the mice with both FCGR2B and STING defects restores the lupus phenotype (115). MYSM1 interacts with STING and cleaves the ubiquitination of the STING at Lys63 to inhibit cGAS-STING signaling (**Figure 7A**) (116). In PBMCs from patients with SLE, the expression of MYSM1 was reduced, while the level of IFN-I and pro-inflammatory cytokines were increased.

During the development of SLE, the mTOR signaling is activated, and blocking the mTOR pathway using rapamycin has emerged as a new strategy for treating SLE in animal models and patients (117–119). A phase 1/2 clinical trial of rapamycin showed the improvement in disease in SLE patients over a 12-month treatment period (120). Inhibition of mTOR by rapamycin prevented IFN-I production by SLE monocytes and promoted autophagy-mediated degradation of STING (121). Transmembrane protein 203 (TMEM203) is an intracellular regulator of STING-mediated signaling that interacts with STING to activate the cGAS-STING signaling pathway (122). The signaling of TMEM203 is elevated in T cells isolated from SLE patients and correlates with disease severity, and inhibiting TMEM203 may also be a potential therapeutic option for the treatment of SLE (**Figure 7A**) (123).

Although both SLE elicitation and development appear to be associated with STING and IFN-I upregulation, there are contradictory results in different mouse models of lupus. It suggested that STING could also be a negative regulatory factor in SLE. The deficiency of STING failure to constrain aberrantly activated TLR signaling cascades responsible for the disease (124). In addition, the knockdown of IFNAR in MRL/LPR mice exacerbates lymphocyte proliferation, autoantibody production, and organ damage (125, 126). Therefore, the role of STING in the pathogenesis of SLE still needs to be further investigated.

#### 4.3.2 Rheumatoid arthritis

The pathogenesis of RA is associated with dsDNA accumulation (127). Deoxyribonuclease II (DNase II) can degrade DNA by hydrolyzing its phosphodiester bonds to prevent its abnormal accumulation. The lack of DNase II prevents this process and promotes STING activation and IFN-dependent systemic auto-inflammation, such as collagen-induced arthritis (CIA) (**Figure 7B**) (128, 129). STING gene-deficient mice had significantly higher levels of anti-collagen antibodies and showed better survival rates than wild-type (WT) mice (128). STING promotes the expression of IFN-inducible genes and the expansion of dendritic cells in CIA. In the CIA model, STING plays a negative regulatory role in B cells when BCR is involved. The inhibition of STING promoted anti-collagen antibody production and B-cell survival, and STING-deficient mice did not spontaneously develop similar autoimmune symptoms (7).

#### 4.3.3 Sjögren’s syndrome

SS is a chronic autoimmune disease affecting multiple organ systems and is characterized by elevated IFN-I levels, which have likewise been reported to be associated with STING (130). Subcutaneous administration of DMXAA to female C57BL/6 mice induced features similar to those of SS patients, such as hypoglandular function and autoantibody production. Activation of STING induced an increase in the expression of IFN-β, IL-6, TNF-α, and IFN-γ in salivary glands and the recruitment of type 1 innate lymphoid cells (ILC1) to the lungs, thereby causing persistent inflammation in the lung (131).

## 5 Inhibitors targeting cGAS-STING-TBK1 axis

### 5.1 Inhibitors targeting cGAS

#### 5.1.1 Catalytic site cGAS inhibitors

**Figure 8:**
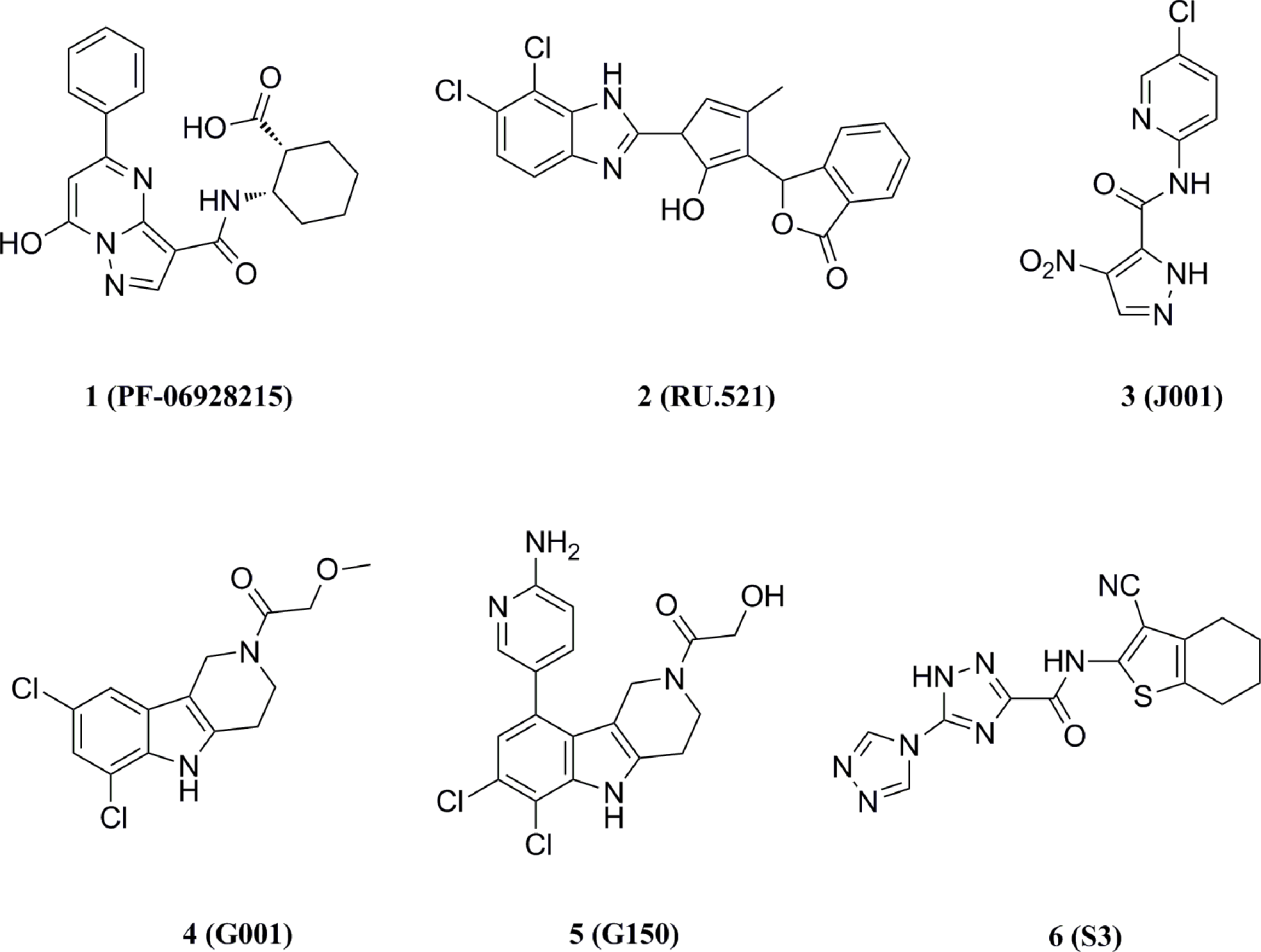
Structures of cGAS inhibitors targeting catalytic site.

Hall et al. performed a saturation transferred differential 1H NMR screening for the Pfizer fragments library using the cGAS crystallized structure, and a low-affinity fragment tetrazolo[1,5-a]pyrimidine (Kd = 171 μM) was identified with weak inhibition of cGAS (IC50 = 78 μM) (132–134). Further optimization for this compound led to compound 1 (Kd = 0.2 μM, IC50 = 4.9 μM) (**Figure 8**). However, compound 1 lacked inhibitory activity in cellular assays for high levels of intracellular ATP and GTP.

Moreover, Vincent et al. performed a high-throughput screen of 123306 compounds and identified four compounds that exhibited good activities (135). The compounds occupied the active center of mouse cGAS and formed key stacking interactions with Agr376 and Try436 at the catalytic site. Based on the binding mode, they subsequently obtained the high-affinity cGAS ligand 2 (Kd = 36 nM) with the best inhibitory activity in cellular assays (IC50 = 0.70 μM) (**Figure 8**). Meanwhile, the tested results in other signaling pathways showed that compound 2 was a selective inhibitor of cGAS and reduced the mRNA level of IFN-β in bone marrow derived macrophages (BMDM) of AGS model TREX1-/- mice, thus indicating the potential for the treatment of autoimmune diseases.

Lama et al. performed an ATP-coupled high-throughput assay for the identification of small molecule inhibitors of h-cGAS (136). Two cross-species active compounds, 3 and 4 (G chemotype backbone), were obtained after multiple rounds of screening (**Figure 8**). Their analogue compound 5 exhibited the good inhibitory activity against both THP-1 cells (IC50 = 1.96 μM) and primary human macrophages (IC50 = 0.62 μM) (**Figure 8**). Compound 5 showed selective inhibition of cGAS in a series of inhibition tests of other innate immune pathways. The structural biology data identified its analogs binding to the cGAS active site. However, the G backbone compounds do not fully occupy the ATP and GTP binding pockets of cGAS and fail to give a clear structure-activity relationship (SAR), and further optimization studies on this backbone are still required.

**Figure 9:**
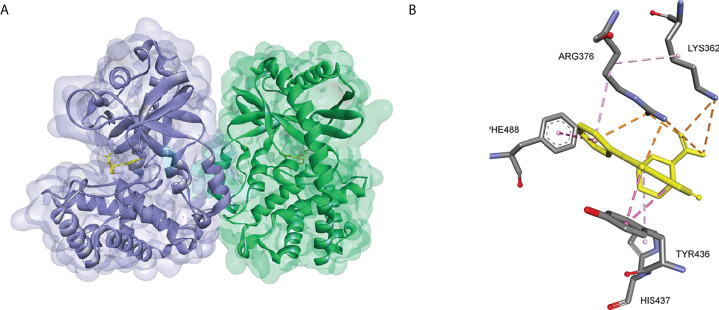
**(A)** Crystal structure of the cGAS inhibitor PF-06928215 bound to a cGAS dimer (PDB-ID: 4LRC); **(B)** Residues of the cGAS active center interacting with PF-06928215 and the interactions (PDB-ID: 4LRC).

The crystal structures of cGAS have been solved, which provides quite useful information for structural-based drug design. Based on the high-resolution crystal structure (1.8 Å) of cGAS and compound 1 complex (**Figure 9**), four effective fragments were identified by virtual screening and thermal shift analysis by Zhao et al. (137) Subsequently, the inhibitory activity of 59 compounds was evaluated using PPiase-coupled assays. One of these compounds did not show any activity in the thermal shift assay and was found to have better inhibitory activity. A similarity search based on this compound was performed and compound 6 (IC50 = 4.9 μM) was identified by PPiase coupling assay (**Figure 8**).

#### 5.1.2 cGAS inhibitors interpret DNA-cGAS interaction

**Figure 10:**
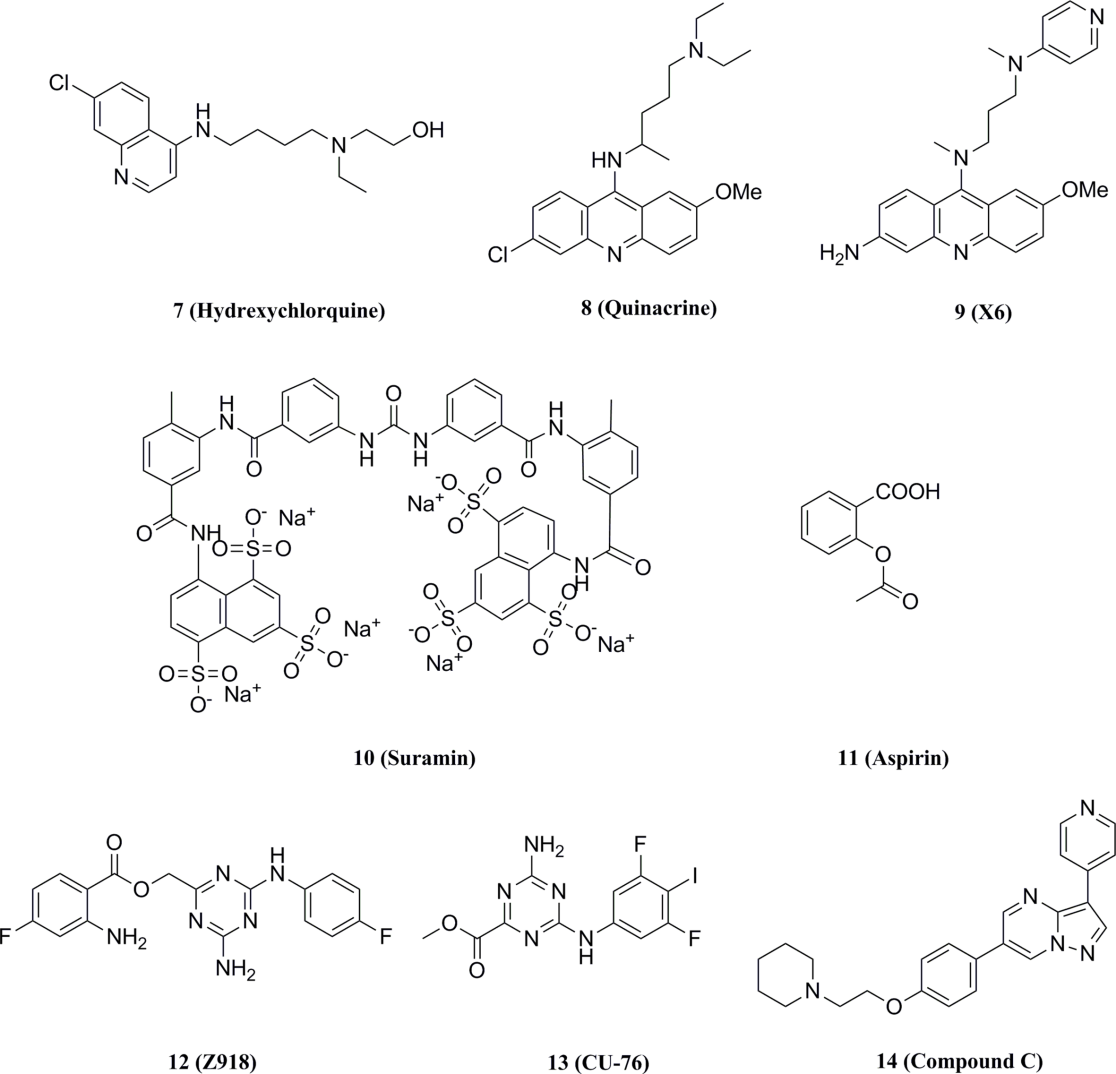
Structures of other types of cGAS inhibitors.

Anti-malarial drugs such as compound 7 and compound 8 have been reported to disrupt the binding of cGAS to dsDNA and selectively block cGAS-double-stranded β interactions to inhibit the IFN expression (**Figure 10**) (138, 139). In turn, a second-generation compound 9 was obtained and the potential for this type of compounds in therapy of autoimmune diseases such as AGS or SLE was validated in the AGS model TREX1-/- mice (**Figure 10**) (140).

Wang et al. screened a library of 268 compounds to obtain the cGAS inhibitor Suramin, which interfered with the formation of the cGAS-dsDNA complex by competing with the dsDNA binding site of cGAS (**Figure 10**) (141). It was also proposed that the potential mechanism of action of compound 10 was that its anionic sulfate acted as a phosphate mimetic, binding to the positively charged region on cGAS (138). Besides, Dai et al. found that acetylation of any of the three cGAS residues K384, K394, or K414 affected the binding of cGAS to DNA (142). Further studies revealed that compound 11 which might acetylate the residues significantly reduced ISGs in peripheral blood mononuclear cells of AGS patients and attenuated the auto-DNA-induced autoimmune symptoms in TREX1-/- mice (**Figure 10**).

#### 5.1.3 cGAS inhibitors with the undisclosed mechanism

Padilla-Salinas et al. performed a virtual screen towards a potentially druggable pocket around Lys347 and Lys394 in h-cGAS to develop protein-protein interface inhibitors of the cGAS dimer itself (143). Only one hit (12) was active to inhibit h-cGAS in vitro with the IC50 of 100 μM (**Figure 10**). Subsequent optimization resulted in a highly potent inhibitor 13 (IC50 = 0.24 μM) which selectively inhibited the activity of cGAS (**Figure 10**). Molecular docking suggested that this series of compounds might bind to a binding pocket other than the dsDNA binding site or the catalytic site, while the crystal structure of compound 13 and the cGAS complex could not be solved to verify the precise binding mode. In addition, Aduro Biotech has disclosed several classes of cGAS inhibitors which have shown a good inhibitory activity at both protein and cell levels. However, the underlying mechanism of cGAS inhibition by these compounds is still required for further elucidation (144–146).

In addition, an oligonucleotide A151 was reported to inhibit cGAS activity (147). A151 contains four TTAGGG motif repeats that can act as an inhibitor of cGAS by interacting with the dsDNA binding domain. In cellular experiments, A151 effectively abolished the activation of cGAS by cytoplasmic DNA, thereby inhibiting the production of IFN-I by human monocytes and preventing endogenous DNA accumulation in TREX1-deficient monocytes. The inhibitory activity of A151 is dependent on the nucleotide sequence and phosphate backbone structure, but its specific binding site to cGAS remains to be further explored (148). Through a screen of 2’OMe ASOs and further sequence mutant, Valentin et al. recently characterized key features within the 20-mer ASOs regulating cGAS and TLR9 inhibition and identified a highly potent cGAS inhibitor, which exhibited more potently than A151 (149).

As a potent inhibitor of AMPK, Lai et al. found that compound 14 also inhibited dsDNA-dependent induction of IFN-I (150–152). Further experiments showed that IFN-β was down-regulated by compound 14 through the inhibition of cGAS rather than the inhibition of STING or TBK1. The IFN-β expression was also inhibited by compound 14 in knockdown AMPK cell lines. Subsequent experiments showed that compound 14 improved the autoimmune phenotype of TREX-/- cells. However, they did not resolve the crystal structure of the complex formed by compound 14, cGAS, and dsDNA. They suggested that compound 14 did not bind directly to the cGAS active site, but rather inhibited the upstream genes of the cGAS-mediated pathway. Besides, Huffman et al. develop a stereoselective butyrolactone coupling with the rapid construction of C-C bonds (153). By this method, four inhibitors for chemical screening of cGAS-STING pathway-targeted cell phenotypes were identified based on a 250,000 compound library.

Besides, cGAS binds DNA in a sequence-independent manner through multivalent interactions mediated by its catalytic core and its positively charged disordered N-terminal domain and induces liquid-liquid phase separation (LLPS) of cGAS-DNA bimolecular condensates (154, 155). Recent works indicated that the natural product epigallocatechin gallate (EGCG) directly impacted DNA-induced cGAS-LLPS in vitro, which might represent a novel opportunity to control some self-autoimmune diseases driven by cGAS (156, 157).

### 5.2 Inhibitors targeting TBK1

#### 5.2.1 BX795 aminopyrimidine-like small molecular TBK1 inhibitors

**Figure 11:**
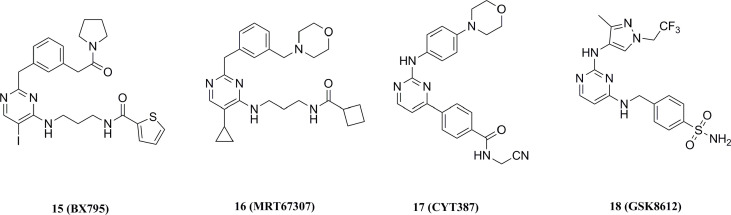
Aminopyrimidine structural TBK1 small molecular inhibitors.

15 (IC50 = 6.0 nM) was the earliest TBK1 inhibitor reported in 2009 (**Figure 11**) (158). This compound was originally developed as an inhibitor of 3 phosphoinositide-dependent protein kinase 1 (PDK1, IC50 = 111 nM), but has also shown strong inhibition of several other kinases (136). Biological assays had shown that 15 inhibited the inflammatory response induced by gram-positive bacteria and the infection of cells with multiple drug-resistant strains of herpes simplex virus type 1. In addition, 15 inhibited the proliferation of oral squamous cell carcinoma (OSCC) by inducing apoptosis and M-phase blockade (159). However, off-targeting effects of 15 on other kinases limited its further development. Further optimization of 15 resulted in 16 (TBK1 IC50 = 19.0 nM, IKKϵ IC50 = 160.0 nM), which showed good selectivity for IKKα, IKKβ, etc (**Figure 11**) (160). The co-X-ray crystal structure of TBK1 with 16 shows that it binds to TBK1 in a similar pattern but forms fewer interactions with the kinase compared to 15, resulting in reduced potency and off-target effects (37).

Likewise, the JAK1/2 kinase inhibitor 17 for the treatment of myelofibrosis exhibited inhibitory activity for TBK1 (IC50 = 58 nM, **Figure 11**) (161, 162). 18 is also a highly selective TBK1 inhibitor (pIC50 = 6.8), and this compound effectively inhibits TBK1-mediated IRF3 phosphorylation and IFNα/β production in addition to its high water solubility and cell permeability (**Figure 11**) (163). Since all of these early TBK1 inhibitors carry a central aminopyrimidine backbone, SAR studies based on this backbone will further deepen the understanding of the pharmacophores for such type of TBK1 inhibitors.

#### 5.2.2 Amlexanox and its derivatives

**Figure 12:**
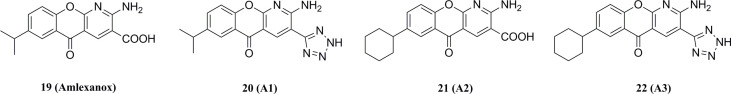
Amlexanox and its derivatives.

19 (TBK1 IC50 = 0.8 μM, IKKε IC50 = 5.8 μM) is a drug approved for the treatment of mouth sores and asthma. Biological studies have shown that 19 increases energy expenditure by increasing thermogenesis, improving insulin sensitivity, and reducing body weight and steatosis in mice (**Figure 12**) (164, 165). In addition, 19 has been found to alleviate acetaminophen-induced liver fibrosis and acute liver injury in mice by inhibiting TBK1/IKKε (166). However, the low solubility and moderate potency of 19 limited its further development. Further structural modifications were performed to the C3-carboxylic acid and C7-isopropyl substituents of 19. Among the analogs, only the tetrazole-substituted compound 21 containing C3-carboxylic acid showed strong inhibition of TBK1 (IC50 = 0.4 μM) and IKKε (IC50 = 0.2 μM), but the cellular activity of this compound was low (**Figure 12**). Among the other analogs, C7-cyclohexyl analog 22 produced the highest levels of IL-6 secretion in 3T3-L1 cells, but none of these compounds had a synergistic effect.

#### 5.2.3 TBK1 inhibitor based on PROTAC technology

**Figure 13:**
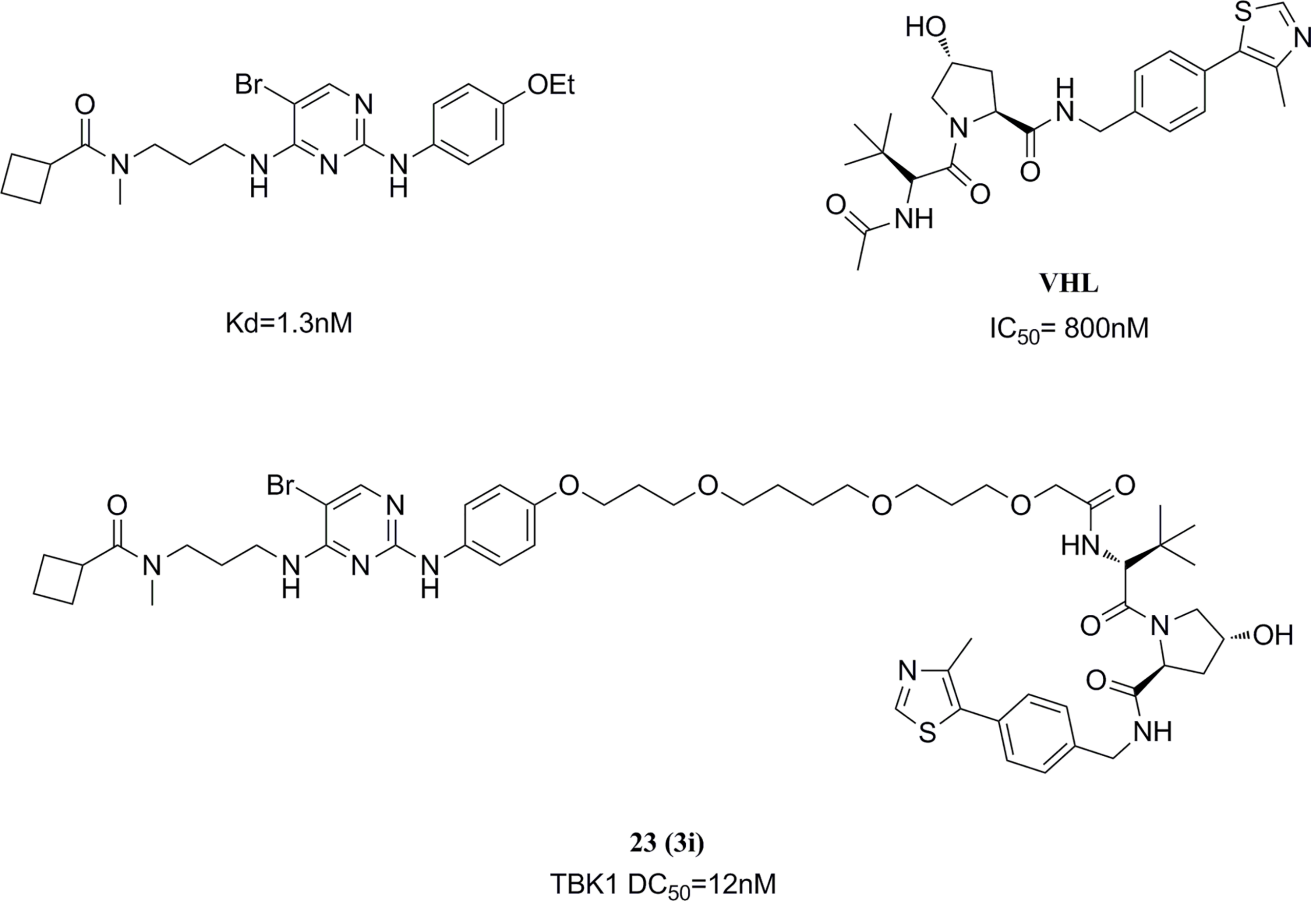
TBK1 targeting PROTAC molecule.

PROTAC (Proteolysis Targeting Chimeras) is an emerging and popular technology in the field of drug discovery in recent years (167). Based on this technique, the Crews group selected a TBK1 bound inhibitor 2,4-diaminopyrimidine-like structure and VHL (Von Hippel Lindau) ligand as a linkage model for PROTAC construction (168). After the optimization, the highly efficient TBK1 inhibitor 23 (TBK1 DC50 = 12 nM, Dmax=96%) was obtained, with good selectivity for the related kinase IKKϵ. (**Figure 13**) The ability of PROTACs to display high potency and selectivity towards TBK1 was revealed by changing the linker length and modulating the binding affinity. The potential for PROTACs was further confirmed in several cancer cells, where TBK1 was almost completely degraded and had no effect on the proliferation of tested cancer cells.

#### 5.2.4 Other small molecular TBK1 inhibitors

**Figure 14:**
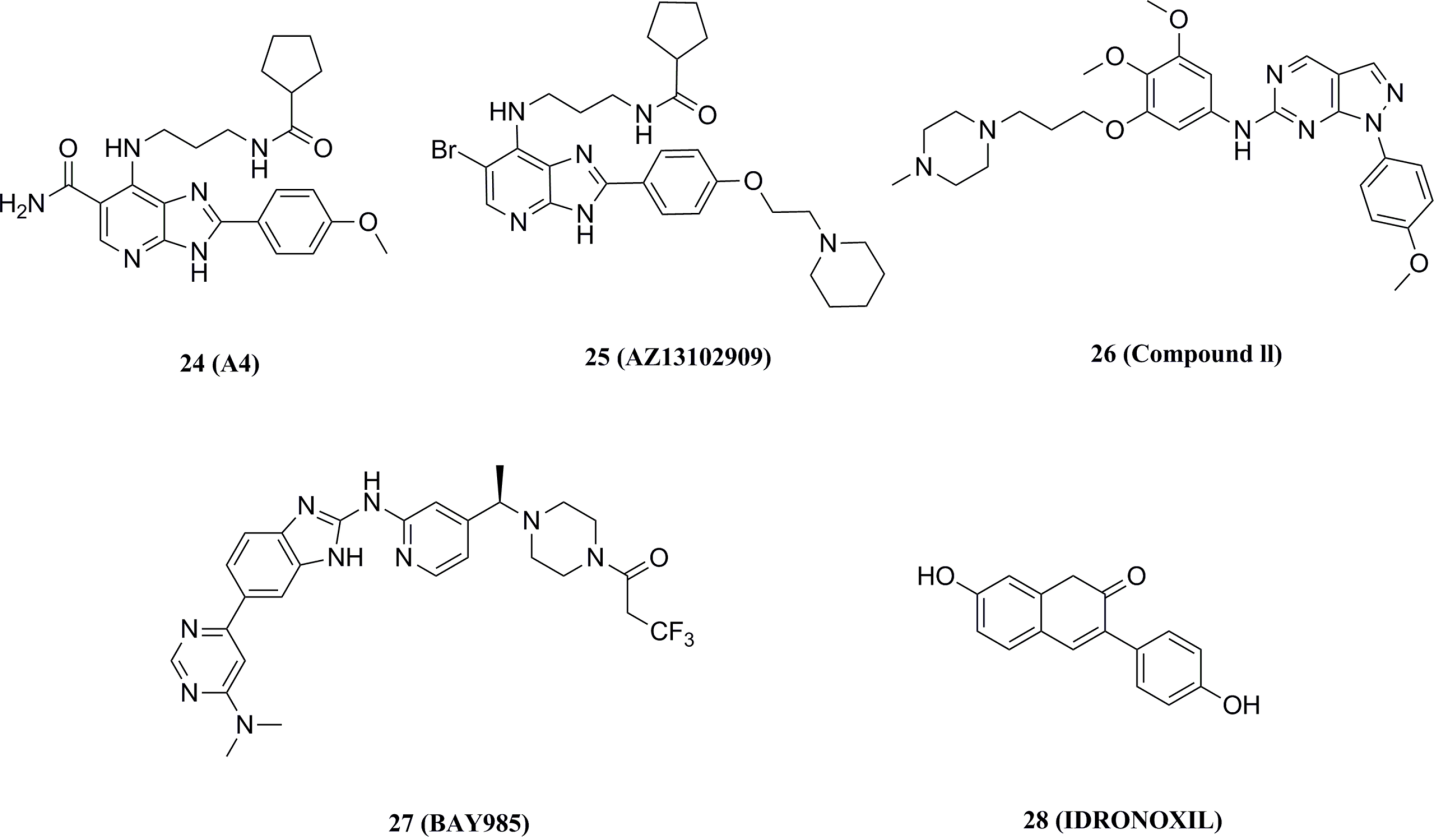
Other TBK1 small molecule inhibitors.

Wang et al. reported a series of imidazopyridines as TBK1 inhibitors, of which the representative compound 24 (IC50 = 9 nM) showed enhanced efficacy and good kinase selectivity (**Figure 14**) (169, 170). The structurally similar imidazopyridine derivative 25 (IC50 = 5 nM) synergized with the MEK inhibitor AZD6244 to induce apoptosis in drug-resistant NRAS-mutant melanoma cells (**Figure 14**) (171). In contrast, 26 (IC50 = 13 nM), also with an imidazopyridine backbone, was found to be a potent, low toxicity inhibitor of TBK1 with promising therapeutic effects in mice against autoimmune diseases such as systemic lupus erythematosus (**Figure 14**) (172). The compound also inhibited the growth of cancer cell lines in non-small cell lung cancer by inhibiting TBK1, thereby leading to a reduction in downstream AKT signaling. The benzimidazole compound 27 (IC50 = 2 nM) reported by Bayer is a highly selective TBK1 inhibitor, but its poor pharmacokinetic properties led it to exhibit poor anti-tumor activity in melanoma mice (**Figure 14**).

Recently, idronoxil 28 is found to be effective in inhibiting the STING signaling pathway. 28 was reported to disrupt the complex formed by TBK1 and STING, blocking the phosphorylation of Ser172 and leading to dual inhibition of the IRF3 and NF-κB transcriptional programs (**Figure 14**) (173). 28 has shown promising results in models for the treatment of COVID-19, providing a potential drug with direct access to the clinic for the treatment of inflammatory diseases.

### 5.3 Inhibitors targeting STING

#### 5.3.1 Competitive inhibitors for CDN binding site

**Figure 15:**
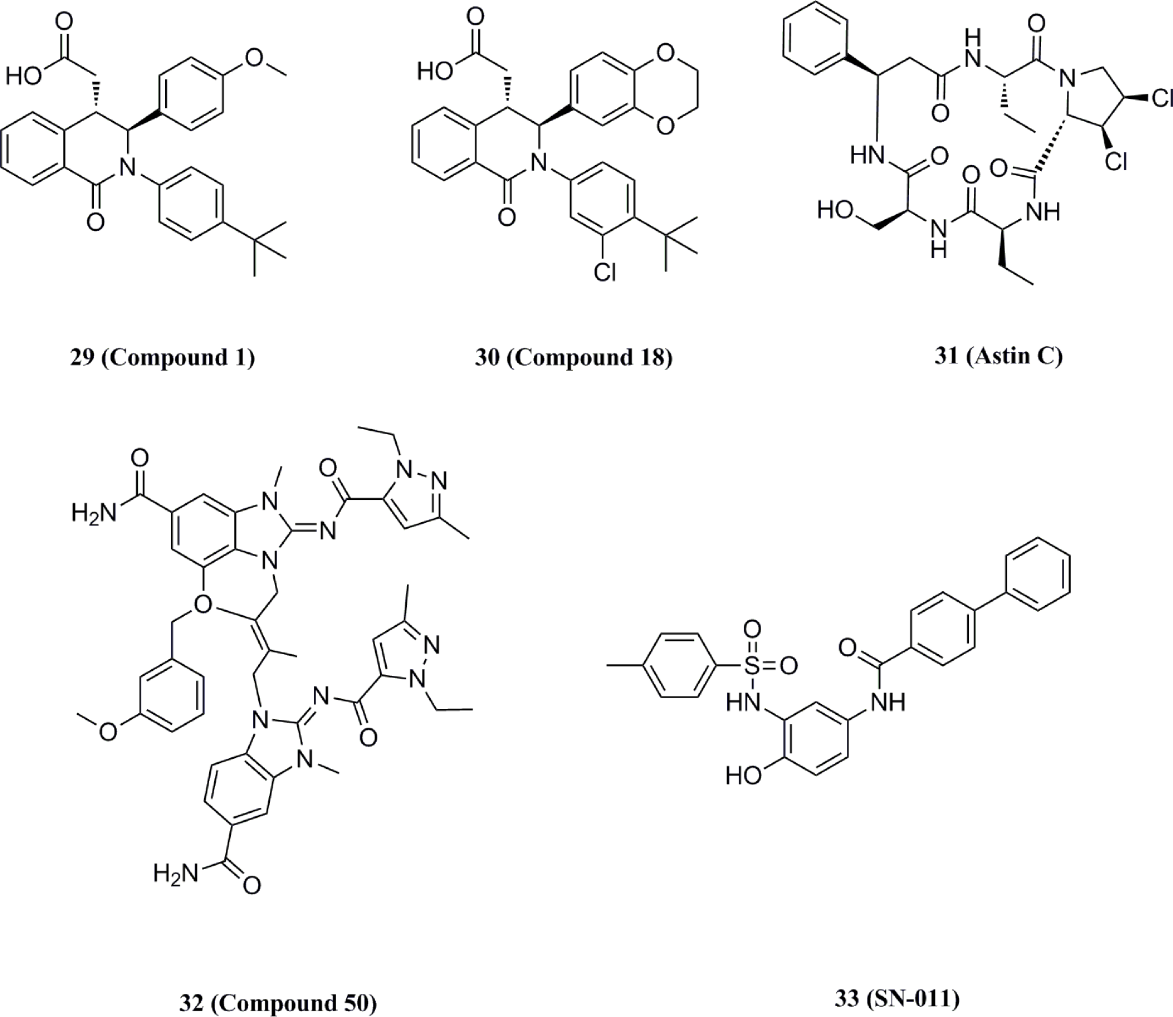
Structures of STING competitive binding antagonists.

**Figure 16:**
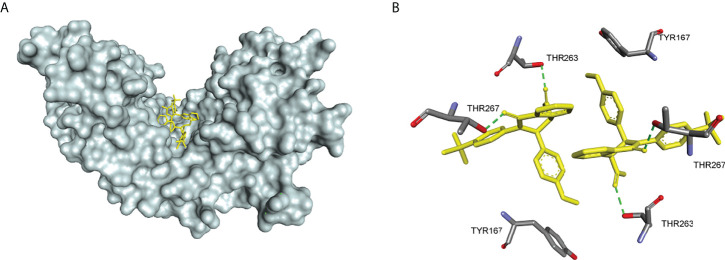
**(A)** X-ray structure of high-affinity ligand bound to STING protein (PDBID: 6MX3); **(B)** the interactions between compound 1 and Thr-263 and Thr-267, green dashed line indicating selected hydrogen bonding interactions.

In 2018, Siu et al. used the symmetry of the CDN binding domain to design small molecular inhibitors that were able to bind to the STING protein. Using mass spectrometry-based ligand screening techniques, they found a low-affinity hit (compound 29, R71H-G230A-R293Q HAQ STING IC50 = 7.3 μM) (**Figure 15**). Co-crystal of determination showed that STING adopted an inactive open conformation, with two molecules occupying the CDN ligand pocket (**Figure 16**). Based on the identification of several major hydrophobic interactions and polar contact between compound 29 and STING protein, compound 30 (HAQ STING IC50 = 0.08 μM) was identified by further SAR studies, which bound to STING similarly and could inhibit 2’3’-cGAMP-induced the secretion of IFN-β with an IC50 of 11 μM (**Figure 15**).

Li et al. identified the natural product 31 from a composite-type cyclopeptides screen based on a reporter gene assay. Further experiments using biotin-labeled compound 31 and h-STING demonstrated the competitive binding of compound 31 to the CDN site, and the addition of high concentrations of CDN (10-fold) abolished the binding of biotin-labeled compound 31 with STING (**Figure 15**) (174). Subsequent mechanistic studies showed that compound 31 locked the recruitment of IRF3 to STING signaling vesicles without affecting the DNA sensing and TBK1 recruitment, thereby preventing the downstream signaling in the cGAS-STING pathway. Notably, GlaxoSmithKline disclosed a series of N-methylamide-based benzimidazole-like STING antagonists in a patent (**Figure 15**) (175). The analogs are derived from the previously reported agonist diABZI, which occupies the CDN site at the STING dimer interface. Notably, compounds in this group, such as compound 32, have shown promising inhibitory properties in both binding assays and cellular experiments (FRET pIC50 > 9.9, THP-1 pIC50 = 8.9%, hPBMC pIC50 = 7.1%) (51).

In 2021, Hong et al. obtain the STING small molecular inhibitor compound 33 (IC50 = 0.076 μM) by the virtual screening towards the STING CDN site and following SAR studies (**Figure 15**) (176). Compound 33 has a higher affinity with binding to the upper CDN binding pocket compared to endogenous 2’3’-cGAMP and locks the STING dimer in an open inactive conformation. This process prevents STING from the oligomerization, translocation, and activation of cytoplasmic DNA, thereby significantly reducing STING-driven IFN-I and pro-inflammatory cytokine expression. Subsequent cellular and animal experiments showed that the compound not only inhibited the over-activation of STING mutants from SAVI patients but also significantly alleviated auto-inflammatory symptoms and prevented the death in TREX1-/- mice. Meanwhile, this compound exhibited comparable inhibitory activity to the previously reported STING covalent inhibitor compound 36 without cytotoxicity, which provides strong support for the development of STING inhibitors for the treatment of STING-related autoimmune diseases.

#### 5.3.2 Covalent inhibitors

**Figure 17:**
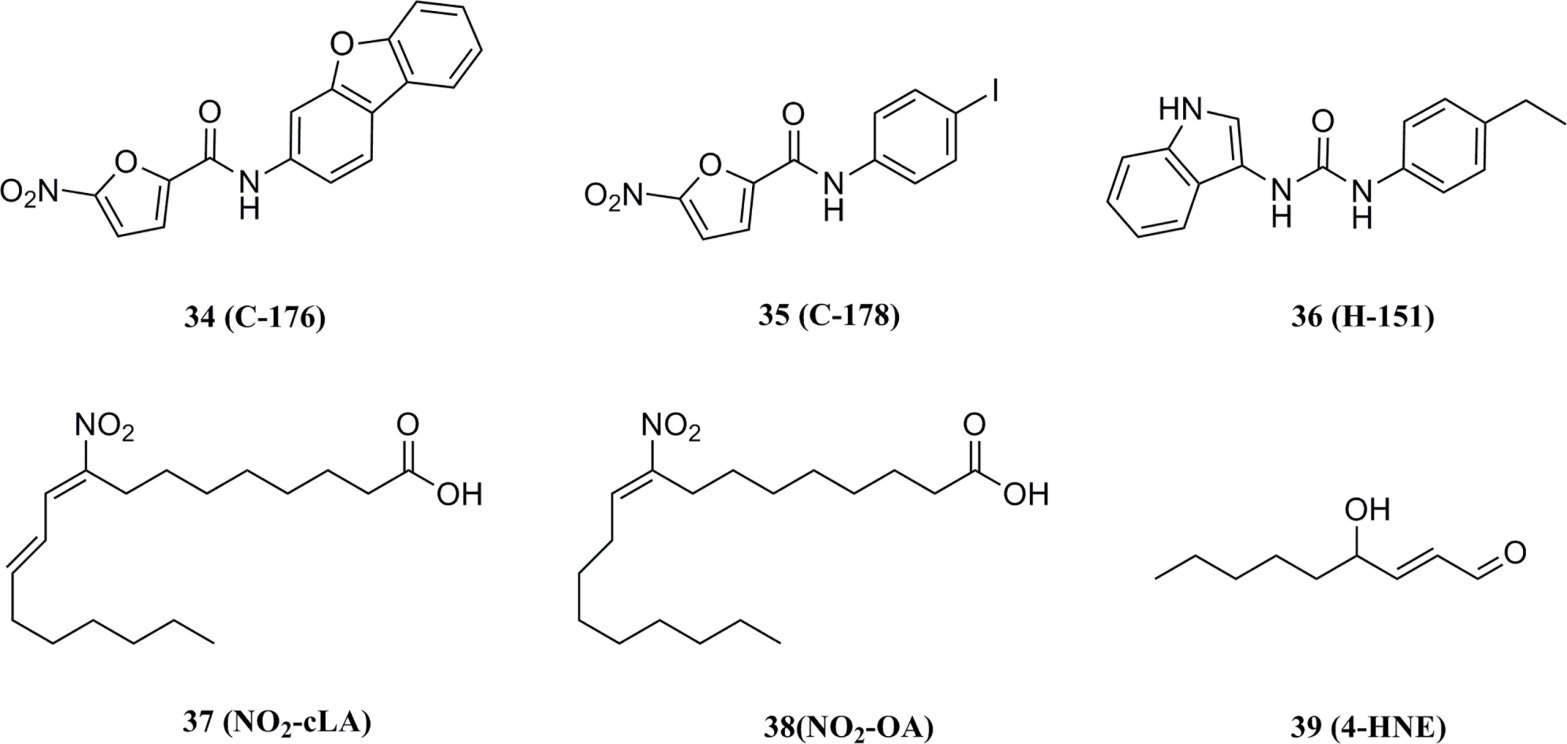
Structures of STING covalent binding antagonists.

**Figure 18:**
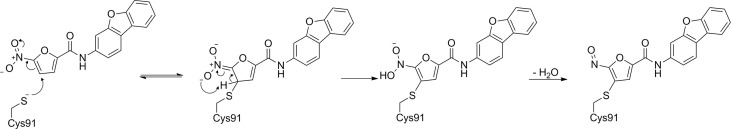
Possibly reaction mechanism of covalent small molecular inhibitors and STING proteins (using C-176 compound as an example).

In 2018, Haag et al. discovered the covalent STING inhibitor compound 36 through structural optimization based on the structures of compound 34 and compound 35, the mouse STING covalent inhibitors which were obtained through high-throughput screening (**Figure 17**) (177). Compound 36 and the analogs blocked the activated palmitoylation of STING by covalently binding to Cys91, thus preventing STING from assembling into a multimeric complex in the Golgi apparatus, thus inhibiting its downstream signaling (**Figure 18**). Importantly, compound 36 shows great potential for the treatment of autoimmune diseases. Compound 36 significantly reduced the systemic cytokine response in CMA-treated mice. Treatment with compound 36 in ALS-model mice effectively ameliorated the inflammatory signal caused by the accumulation of TDP-43, restoring neuronal number and motor function (17, 178). Subsequently, compound 36 was found to reduce the symptoms of the chronic inflammatory disease psoriasis by decreasing protein levels of the pro-inflammatory cytokines IL-17A, IL-23, and IL-6 in serum and skin lesions (22).

Besides, Hansen et al. found that host infection with HSV results in the formation of nitro fatty acids in vivo and that endogenous nitro fatty acids (NO2-Fas) can covalently modify STING by Michael addition reaction to adjacent cysteines at positions 88 and 91 (Cys88/91) or N-terminal histidine (His16), thereby inhibiting the palmitoylation of STING and subsequent production of IFNs in host cells (**Figure 17**) (179). Similarly, the lipid peroxidation during viral infection leads to an increase in one of the major products, 4-hydroxynonenal (38), which promotes the carbonylation of STING, thereby inhibiting the transport of STING from the ER to the Golgi apparatus and suppressing STING activation (**Figure 17**) (180).

#### 5.3.3 PROTAC target STING

**Figure 19:**
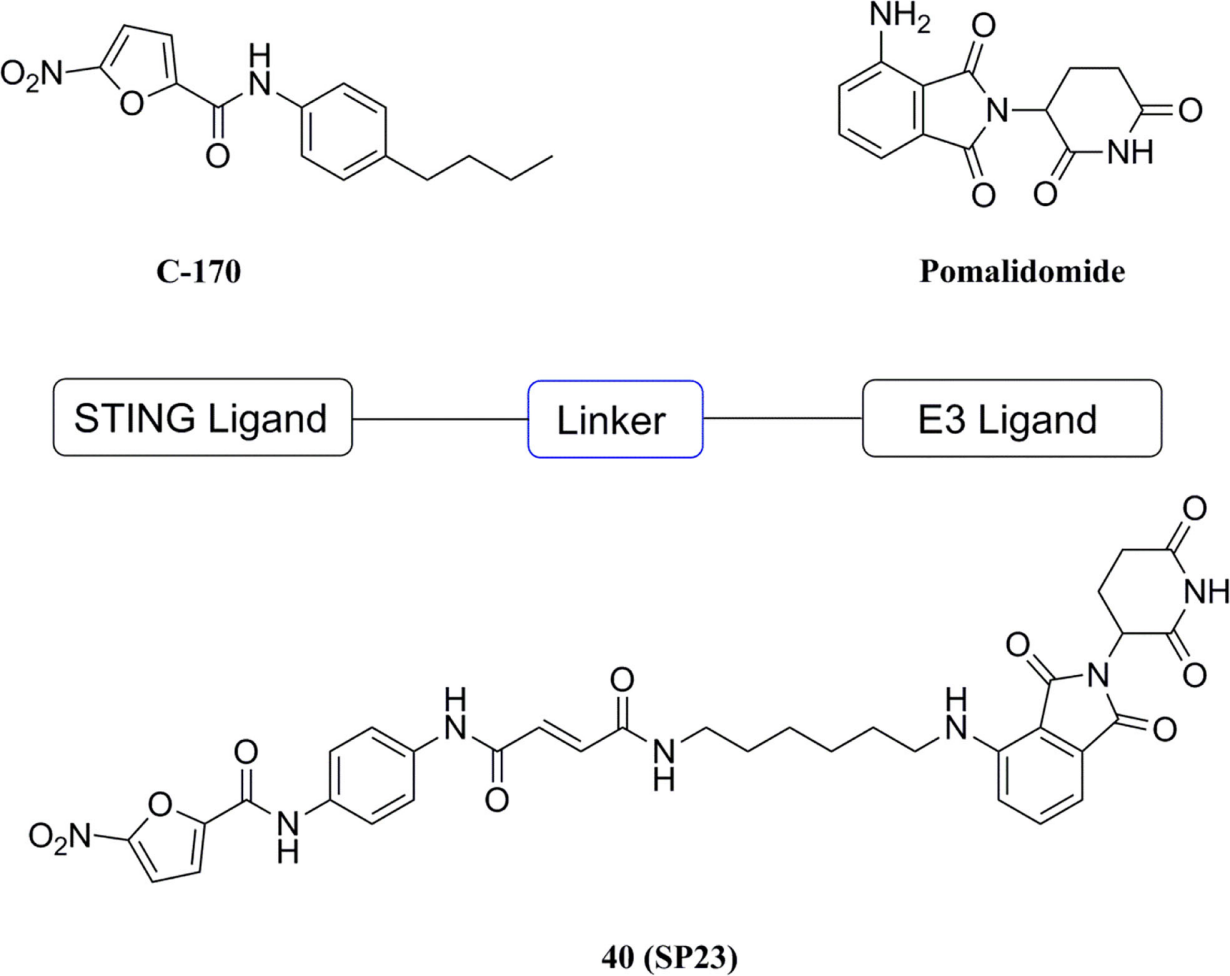
PROTAC molecule target STING.

Based on the PROTAC technique, Liu et al. selected the previously reported STING inhibitor C-170 linked to pomalidomide (CRBN ligand) as the PROTACs targeting STING (**Figure 19**) (181). Among them, compound 40 (DC50 = 3.2 μM) induced the degradation of STING via the CRBN-dependent ubiquitin-proteasome pathway, and dose-dependently downregulated the levels of IFN-β, IL-6, and CXCL10 triggered by 2’3’-cGAMP in THP-1 cells. A partial biological evaluation of this compound as an anti-inflammatory agent was also performed. PROTAC has the advantages of reduced drug exposure, low toxicity, and overcoming drug resistance compared to conventional drugs, and this compound has been reported to provide an alternative strategy for the development of new STING inhibitors.

## 6 Discussion and Perspective

The GAS-STING signaling plays a critical role in the innate immune response, and the abnormal activation of GAS-STING is linked to various autoimmune diseases (182). The genetic mutants which induce the continuous activation of STING or the cytoplasmic dsDNA accumulation contribute to several STING-relevant autoimmune diseases. While for some autoimmune diseases like SLE which are regarded as the systemic disease for multifactorial complex pathogenesis, the GAS-STING signaling activation is only one of the multiple factors. Moreover, quite a few neurodegenerative diseases including ALS and NPC belong to STING-relevant autoimmune diseases. Importantly, the knock-outing of the STING gene would ameliorate the pathological features of the STING-relevant autoimmune disease, which indicate that the cGAS-STING-TBK1 axis is a promising therapeutic target for various autoimmune diseases.

The inhibitors of cGAS-STING-TBK1 signaling were reported to decrease the protein levels of the inflammatory cytokines and the inflammatory signaling at the cellular and animal levels. The covalent STING inhibitor compound 36 significantly decreased systemic cytokine responses in CMA (the STING agonist) treated mice, thereby attenuating symptoms of autoinflammatory disease in vivo. Moreover, another CDN binding STING inhibitor compound 33 treatment shows the comparable suppression of IFN-β and ISGs expression in the TREX1−/−mice. Therefore, the STING antagonists have become the potential therapeutic agents for STING-relevant autoimmune diseases. Besides the direct inhibition of the STING signaling, targeting the upstream and downstream nodes of the STING activation pathway is also an alternative way to develop the drug against autoimmune diseases. For instance, inhibitors targeting cGAS significantly downregulated IFN expression in TREX1-/- PMBCs in AGS model mice, showing excellent potential for drug development in the treatment of autoimmune diseases caused by DNA accumulation such as AGS and SLE. However, drugs targeting cGAS did not show promising results in the SAVI model, and treatment of cGAS-independent autoimmune diseases may still have to focus on small molecular inhibitors targeting STING. Nerveless, cGAS inhibitors still can be a good complement to therapeutic regimens for the treatment of DNA-dependent autoimmune diseases. Besides, it was reported that TBK1 inhibitor BX795 could downregulate IFN-I activation in PBMCs of SS, SLE, and MS patients (183, 184). Compared to blocking common natural immune targets, such as TBK1 or IFNs, inhibition of cGAS-STING has less risk of immunosuppression and opportunistic infections without keeping the other PRR systems intact. Moreover, STING inhibitors may also be more potent than existing therapeutic agents (e.g., JAK inhibitors and IFN receptor antibodies) because the latter do not limit the maladaptive effects of other cytokines such as TNF-α and IL-6 (185).

Three types of STING inhibitors have been reported including covalent inhibitors forming specific covalent linkage to Cys91, Cys88/91, or His16, with compound 36 as the representative compound (177). The second of type inhibitors just like 28 can disrupt STING/TBK1 interactions (173). The third type of STING antagonists compete with 2’3’-cGAMP at the STING CDN binding site such as 33 (176). Currently, both the first type and the third type show much more potent activity (the IC50 is at the nanomolar level) than the second type inhibitor (the IC50 is at the micromolar level). It was reported that 33 had lower cytotoxicity and higher specificity than compound 36, which indicated that the competed inhibitor at CDN binding site would lead to better specificity and less toxicity. Interestingly, compound 32 which is derived from the STING agonist diABZI exhibits the potent inhibitory of cGAS-STING signaling. The molecular dynamics studies should be performed to investigate how the structural modification in such a scaffold affects the conformational changes of STING CTD and lead to the agonistic or antagonistic activity of the diABZI analogs, which would shed light on the modulation mechanism of STING activation. Moreover, the crystal structures of the complexes of STING and the antagonists have been solved (PDB code: 6MX3), which would facilitate rational drug design based on the complex structure. In fact, the discovery of compound 33 was achieved through the optimization of the active hit which was obtained via the molecular docking towards the CDN site using the virtual chemical database (ZINC).

## Author contributions

MZ, YZ, XZ collect and analysis the reference data. MZ designed the experiments and wrote the original manuscript. JZ reviewed and edited the manuscript. All authors contributed to the article and approved the submitted version. All authors contributed to the article and approved the submitted version.

## Funding

This work was in part supported by the National key research and development program (2018YFE0107600, ZJM), Nature Science Foundation of China (22077115, 81672559). National Mega-Project for Significant new drug discovery (2018ZX09711003-002-002, ZJM).

## Conflict of interest

The authors declare that the research was conducted in the absence of any commercial or financial relationships that could be construed as a potential conflict of interest.

## Publisher’s note

All claims expressed in this article are solely those of the authors and do not necessarily represent those of their affiliated organizations, or those of the publisher, the editors and the reviewers. Any product that may be evaluated in this article, or claim that may be made by its manufacturer, is not guaranteed or endorsed by the publisher.

## Glossary

**Table:**

PAMPs	pathogen-associated molecular patterns
DAMPs	damage-associated molecular patterns
RIG-I	retinoic acid-inducible gene I
MDA5	melanoma differentiation-associated gene 5
TLRs	Toll-like receptors
2'3'-cGAMP	2'3'-cyclic GMP-AMP
ATP	adenosine triphosphate
GTP	guanosine triphosphate
ISGs	interferon-stimulator genes
AGS	Aicardi-Gouti&egrave;res syndrome
SLE	systemic lupus erythematosus
SAVI	STING-associated vasculopathy of infancy
ALS	amyotrophic lateral sclerosis
ER	endoplasmic reticulum
KD	N-terminal kinase domain
ULD	ubiquitin-like domain
SDD	alpha-helical scaffold dimerization domain
NTD	N-terminal transmembrane structural domain
CTD	C-terminal structural domain
LBD	ligand-binding domain
TBM	TBK1-binding motif
CDG	cyclic-di-GMP
CDA	cyclic-di-AMP
COPII	coat protein complex II
TBK1	tank-binding kinase 1
IFNs	type I interferons
IRF3	interferon regulatory factor 3
CTT	C-terminal tail
JAKs	Janus kinases
TYK2	tyrosine kinase 2
STAT1	signal transducer and activator of transcription 1
IRF9	interferon regulatory factor 9
AMPK	AMP-activated protein kinases
mTOR	mammalian targets of rapamycin
ATG9a	autophagy-associated protein 9a
NLRC3	NOD-like receptor C3
WIPI2WD	repeat structural domain phosphoinositides interacting protein 2
RAB7	RAS-associated protein Rab-7a
COPI	coat protein complex I
NF-kB	nuclear factor kappa B
LIRs	LC3 interacting regions
SAMHD1	histidine-aspartate domain-containing protein 1
MRE11A	meiotic recombination 11 homolog A
IFNAR	IFN-αreceptor
STIM1	stromal interaction molecule 1
AD	Alzheimer's disease
PD	Parkinson's disease
HD	Huntington's disease
FTD	frontotemporal dementia
MS	multiple sclerosis
VDAC1	voltage-dependent anion channel 1
C9orf72	chromosome 9 open reading frame 72
NPC1	Niemann-Pick C1
EAE	experimental autoimmune encephalomyelitis
BBI	Bowman-Birk inhibitor
AdMVs	apoptosis-derived membrane vesicles
TMEM203	Transmembrane protein 203
DNase II	Deoxyribonuclease II
CIA	collagen-induced arthritis
WT	wild type
ILC1	type 1 innate lymphoid cells
SAR	structure activity relationship
LLPS	Liquid-liquid phase separation
EGCG	epigallocatechin gallate
PDK1	3 phosphoinositide dependent protein kinase
MLK1-3	mixed lineage kinase 1-3
MARK1-4	AMP-activated protein kinase 1-4)
OSCC	oral squamous cell carcinoma
NSCLC	nonsmall-cell lung cancer
PDAC	pancreatic ductal adenocarcinoma
BMDM	bone marrow derived macrophages
